# Extraorganic Hepatic Artery Aneurysm: Failure of Transcatheter Embolization

**DOI:** 10.1155/1998/34027

**Published:** 1998-08

**Authors:** Pavel G. Tarazov, Vladimir K. Ryzhkov, Vladimir N. Polysalov, Kyrill V. Prozorovskij, Alexej A. Polykarpov

**Affiliations:** 1 Division of Angio/Interventional (P.G.T., V.K.R., A.A.P.) Department of Surgery (V.N.P.) Department of Diagnostic Radiology (K.V.P.) St. Petersburg Research Institute of Roentgenology & Radiation Therapy St. Petersburg Russia; 2 Res Inst Roentgenol ul Leningradskaja 70/4 Pesochny-2 St. Petersburg 189646 Russia

## Abstract

Hepatic artery aneurysm (HAA) was diagnosed in a 62-year-old man who was a poor candidate for surgery because of severe liver cirrhosis and diabetes mellitus. Two attempts to occlude the HAA by transcatheter embolization failed because of recanalization of the aneurysm. Moreover, aneurysmal dilatation of the superior mesenteric artery and the left renal artery developed and progressed. Both the literature and the present case show that an individual approach to treatment of extraorganic HAA should be chosen in dependantan location and anatomy of the lesion.


*HPB Surgery*, 1998, Vol. 11, pp. 55-60
Reprints available directly from the publisher
Photocopying permitted by license only

© 1998 OPA (Overseas Publishers Association) N.V.
Published by license under
the Harwood Academic Publishers imprint,
part of The Gordon and Breach Publishing Group.
Printed in India.

*Case Report*

### Extraorganic Hepatic Artery Aneurysm: Failure of Transcatheter Embolization

PAVEL G. TARAZOV*, VLADIMIR K. RYZHKOV, VLADIMIR N. POLYSALOV, KYRILL V. PROZOROVSKIJ and ALEXEJ A. POLYKARPOV

*Division of Angio/Interventional (P.G.T., V.K.R., A.A.P.), Department of Surgery (V.N.P.), Department of Diagnostic Radiology (K.V.P.), St. Petersburg Research Institute of Roentgenology & Radiation Therapy, St. Petersburg, Russia*

*(Received 14 October 1996; In final form 27 March 1997)*

**Hepatic artery aneurysm (HAA) was diagnosed in a 62-year-old man who was a poor candidate for surgery because of severe liver cirrhosis and diabetes mellitus. Two attempts to occlude the HAA by transcatheter embolization failed because of recanalization of the aneurysm. Moreover, aneurysmal dilatation of the superior mesenteric artery and the left renal artery developed and progressed. Both the literature and the present case show that an individual approach to treatment of extraorganic HAA should be chosen in dependantan location and anatomy of the lesion.**

*Keywords:* Aneurysm, hepatic-embolization, therapeutic

### INTRODUCTION

Hepatic artery aneurysms (HAAs) comprise one fifth of aneurysms affecting splanchnic vessels [1]. The rupture of HAA is a life threatening emergency and is why surgery is recommended in most if not all cases [2]. Transcatheter embolization is usually effective in intrahepatic aneurysms [3] at least in the short term, but may be unsuccessful in extraorganic HAAs [4].

We report a patient with a huge extrahepatic HAA who had very high risk related to surgery. Transcatheter treatment failed to occlude the lesion.

### CASE REPORT

A 62-year-old nonalcoholic man suffering from insulin-depended diabetes mellitus and post-hepatitis B liver cirrhosis (C-12 Child-Pugh's score) was admitted with moderate epigastric and right upper quadrant abdominal pain. He denied any history of abdominal trauma or other disease. There was no bruit or mass in his abdomen. Duplex Doppler ultrasonography showed a rounded hyperchoic mass 90×80 mm in the porta hepatis. The lesion had an intramural tortuous canal of 10 to 20 mm diameter

*Author for correspondence: Res Inst Roentgenol, ul Leningradskaja 70/4, Pesochny-2, St. Petersburg, 189646 Russia.

with arterial flow. The extrahepatic HAA was suspected.

Angiography showed a partially thrombosed aneurysm of the common hepatic artery with involvement of the celiac trunk (Fig. 1a) and also a small aneurysm of the superior mesenteric artery (SMAA). No gastroduodenal artery (GDA) or other collaterals to the liver were seen. Transcatheter embolization was chosen for treatment because of the very high risk of surgery. It was not technically possible to introduce the catheter including small diameter coaxial microcatheter through the aneurysm for embolization of the hepatic artery distal to lesion. Occlusion of HAA canal was performed with 14 Gianturco coils of 5 mm to 10 mm diameter (Cook, Bloomington, IN, USA) (Fig. 1b). There was no complication of the procedure.

Three months later US showed recanalization of HAA just below the previously placed coils. The embolization was repeated with 5 Hilal (Cook, Bloomington, IN, USA) and 5 Gianturco coils.

During the following hospitalization in 6 months, US, computed tomography, and angiography again showed recanalization of HAA canal deeply within the aneurysmal sac (Fig. 1c). Also enlargement of SMAA (Fig. 1d) and aneurysmal dilatation of the left renal artery were seen. It was decided to stop further attempts of transcatheter management.

At present, the patient is alive 3 years after the first admission and receiving medical therapy, with progression of both diabetes and cirrhosis. Subsequent US and computed tomography show no change in aneurysm sizes.

### DISCUSSION

The etiology of HAA includes atherosclerosis, arteritis, trauma and infectious diseases [1, 2, 5]. The less common causes of development of HAA are fibromuscular dysplasia, pancreatitis, syphilis, tuberculosis, and polyarteritis nodosa [6].

In the not too distant past, HAA most often were diagnosed at autopsy, or at times of surgical exploration for major complications of this lesion. The risk of spontaneous rupture

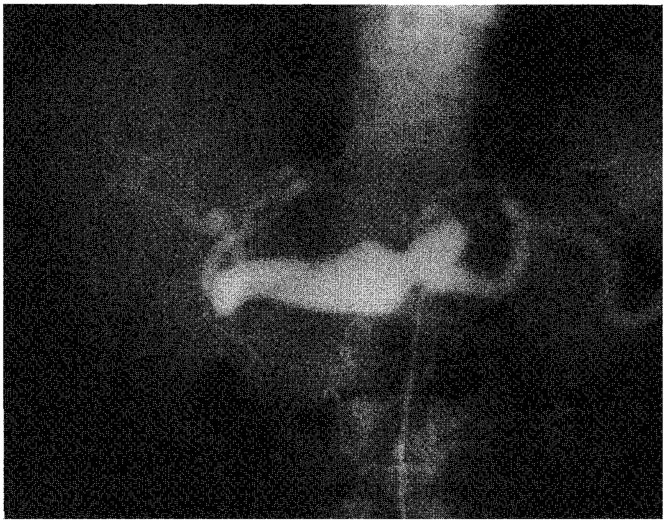

(a)

FIGURE 1a Celiac angiography shows aneurysm of common hepatic artery with occlusion of gastroduodenal artery and partial involvement of celiac trunk. Size of aneurysm according to ultrasonography is noted with arrows.

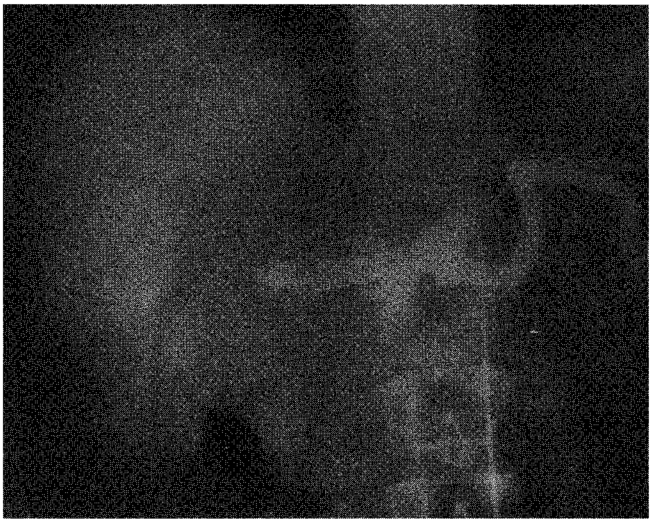

(b)

FIGURE 1b No flow through aneurysm is seen post embolization.

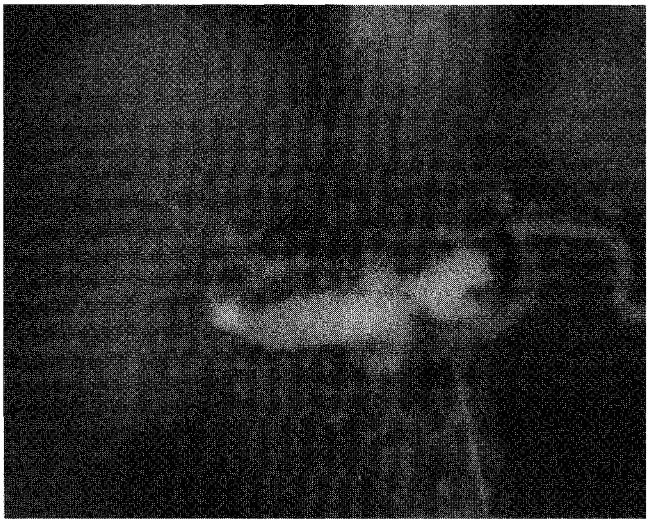

(c)

FIGURE 1c Celiac angiography shows recanalization of hepatic artery aneurysm after two embolization procedures.

of HAA was recognized as 80% before 1960 [2]. With the development of modern imaging techniques, an increasing number of asymptomatic HAAs have been reported, and hemorrhagic complications were documented in less than 20% of patients [1]. So, as more asymptomatic incidental HAAs are diagnosed, the indication for their treatment is more speculative and can be questioned.

There are a few cases in the literature about uneventful follow-up of large extrahepatic HAAs [7, 8]. Our patient had a huge, symptomatic extrahepatic lesion, so surgical or radiologic treatment could be recommended [2, 6, 9].

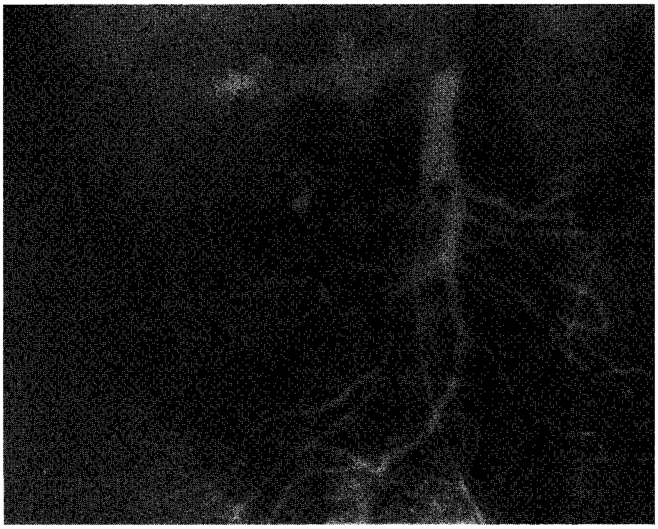

(d)

FIGURE 1d Control angiography shows enlargement of superior mesenteric artery aneurysm from 8 mm to 11 mm in diameter during period between two hepatic embolizations.

Arterial embolization has become the treatment of choice for intrahepatic HAA [1, 3]. The results of transcatheter occlusion of extrahepatic HAAs are contradictory, however. Ballen and Raphael [10] described a patient with the common HAA successfully treated by embolization. Johnson *et al.* [11] performed coil embolization of an aneurysm involving both the gastroduodenal and the common hepatic arteries with a good result. O'Connor *et al.* [12], Cotroneo *et al.* [12] reported on two cases of partial recanalization of previously embolized extrahepatic HAA, but one to six year follow-up showed no hemorrhagic complications in these patients. On the other hand, unsuccessful and complicated outcomes of similar treatment have been reported by other authors [4, 13].

We considered several treatment options in our patient. The risk of major operation (vessel reconstruction or liver transplantation with simultaneous removal of HAA) had unacceptably high risk in this case. Moreover, radicality of surgery would be incomplete because of the presence of other aneurysms [2]. Arterial ligation was more invasive than embolization. Direct puncture and occlusion of extrahepatic HAA was possible [14], but that treatment had a risk of intraperitoneal bleeding [15]. Transluminal stenting of HAA is very perspective: O'Connor *et al.* [12] described an excellent result of the common HAA treatment with Strecker stent. The use of covered stents for management of arterial aneurysmal diseases will soon be available. On the other hand, effectiveness of stents for treatment of aneurysms is still not fully approved. Moreover, improvement of geometry of available stents and successful catheterization of the distal part of HAA (we were unable to do it in our patient) will be needed for this procedure.

Taking these data into account, we decided to perform transcatheter embolization. The standard technique includes occlusion of both outflow and inflow vessels, and packing the aneurysm itself and any neck to the aneurysm [3]. Unfortunately, this technique was not possible in our case. The patient had HAA with suboptimal prognostic features for embolization in that it was fusiform, involved a long segment of the common hepatic artery, and had poor collaterals. In addition, the internal lumen of the aneurysm was relatively small compared to HAA itself. On the other hand, embolization was the only possible low-risk method of treatment for that patient.

After two attempts, further embolization procedures were considered to have a low benefit-to-risk ratio because of both the unsuccessful previous treatments and the presence of other arterial aneurysms. We speculated that these lesions involving SMA and the left renal artery had etiology similar to HAA but we could not exclude the probability of their enlargement after arterial flow redistribution caused by hepatic embolization.

In our patient, the clinical result should not be considered absolutely unsuccessful. Displacement of the aneurysmal lumen from periphery to the center of HAA potentially lowers the risk of spontaneous rupture of the lesion [7, 8].

It can be concluded, that individual approach to treatment of extraorganic HAA should be used depending on the location and anatomy of the lesion. The treatment modalities may vary from simple observation to surgery.

### *References*

[1] Stanley, J. C., Wakefield, T. W., Graham, L. M. and Zelenock, G. B. (1987). Ruptured splanchnic artery aneurysms. In: J. J. Bergan and J. S. T. Yao, Eds: Vascular surgical emergencies. Orlando: Grune & Stratton, Inc., pp. 391–400.
[2] Dougherty, M. J., Gloviczki, P., Cherry, K. J., Bower, T. C., Hallett, J. W. and Pairolero, P. C. (1993). Hepatic artery aneurysms: Evaluation and current management. *Int. Angiol.*, **12**, 178–184.
[3] Reid, C., Cameron, D., Simon, T. A., Ives, J. and Hall, J. C. (1992). Selective embolization of intrahepatic aneurysms. *Aust. NZ. J. Surg.*, **62**, 582–584.
[4] Salam, T. A., Lumsten, A. B., Martin, L. G. and Smith, R. B. (1992). Nonoperative management of visceral aneurysms and pseudoaneurysms. *Amer. J. Surg.*, **164**, 215–219.
[5] Aboujaoude, M., Noel, B., Beaudoin, M., Ghattas, G., Lalonde, L., Bui, T. B. and Oliva, V. L. (1996). Pseudoaneurysm of the proper hepatic artery with abdomonal trauma. *J. Trauma*, **40**, 123–125.
[6] Gehling, G. and Balzer, K. (1994). The diagnosis of hepatic artery aneurysm: A review, *Dtsch. Med. Wschr.*, **119**, 701–704 (in German).
[7] Sgroi, G., Stringhi, E., Bergamaschi, E., Ghilardi, G. and Scorza, R. (1994). A case of asymptomatic giant aneurysm of the common hepatic artery. *J. Cardiovasc. Surg.*, **35**, 337–339.
[8] Lukes, P., Wihed, A., Tidebrant, G., Falk, A. and Ortenwall, P. (1994). Angiography of visceral aneurysms. *Eur. Radiol.*, **4**, 75–79.
[9] Cotroneo, A. R., Salcuni, M., Marano, G., Di Stasi, C. (1996). Common hepatic artery complicated aneurysms: Selective embolization with a glueing fluid. *Radio. Med.*, **91**, 492–496 (in Italian).
[10] Balen, F. G. and Raphael, M. J. (1995). Embolization of hepatic artery aneurysm using tungsten coils: Shedding a new light on an old problem. *J. Intervent. Radiol.*, **10**, 141–144.
[11] Jonsson, K., Bjerustad, A. and Eriksson, B. (1980). Treatment of a hepatic artery aneurysm by coil occlusion of the hepatic artery. *AJR*, **134**, 1245–1247.
[12] O'Connor, P. J., Chalmers, A. G., Chennels, P. M. and Lintott, D. J. (1995). The radiologic treatment of hepatic artery aneurysm. *Clin. Radiol.*, **50**, 792–796.
[13] Onohara, T., Okadome, K., Mii, S., Yasumori, K., Muto, Y. and Sugimachi, K. (1992). Rupture of embolized coeliac artery pseudoaneurysm into the stomach: Is coil embolization an effective treatment for coeliac anastomotic pseudoaneurysms? *Eur. J. Vasc. Surg.*, **6**, 330–332.
[14] Fava, M. P., Cruz, F. O., Lastra, M. V., Aguilar, J. G. and Guzman, S. B. (1994). Common hepatic artery pseudoaneurysm secondary to pancreatitis: Direct percutaneous embolization. *Surg. Endosc.*, **8**, 1223–1226.
[15] Herskowitz, M. M., Flyer, M. A. and Sclafani, S. J. A. (1993). Percutaneous transhepatic coil embolization of a ruptured intrahepatic aneurysm in polyarteritis nodosa. *Cardiovasc. Intervent Radiol.*, **16**, 254–256.

### COMMENT

The treatment of visceral artery aneurysms is challenging. This paper illustrates some of those challenges, specifically relating to embolization as a treatment strategy.

The spectrum of visceral aneurysms has changed over the last decade as improved diagnostic imaging reveals asymptomatic aneurysms and smaller aneurysms. The natural history of this group of aneurysms is unknown, and although there remains a risk of catastrophic, potentially fatal, haemorrhage, this is probably much less than 80% quoted in the literature.

Surgery is the traditional treatment for visceral aneurysms. Due to technical difficulties and the associated morbidity of surgery interventional radiological techniques have emerged. Catheter embolization is now a well described technique for treating visceral aneurysms, and is a treatment option in most major centres. However selection criteria are important before opting for embolization. Ideally an aneurysm should be focal with an accessible afferent and efferent artery suitable for embolization. Sacrifice of the artery should be practical (i.e. collateral arterial supply or patent portal vein). These ideals are frequently compromised, particularly in patients unfit for surgery. As a compromise packing the aneurysm sac with coils is an option. However as this case documents a proportion of aneurysms treated this way will recanalize. Unfortunately we do not know the proportion which will recanalize, and similarly we do not know the clinical significance of this.

Technology is moving on. Hopefully covered stents flexible enough to be deployed in the visceral arteries will soon be commercially available and may revolutionise the treatment of this rare but challenging disease.

A. M. Wood
Consultant Radiologist
Department of Radiology
University Hospital of Wales
Heath Park
Cardiff

### COMMENTARY

Extraorganic hepatic artery aneurysm: failure of transcatheter embolization. P. G. Tarazov, V. K. Ryzhkov, V. N. Polysalov, K. V. Prozorovskij, A. A. Polykarov.

This is an interesting case history of a failed treatment of an extrahepatic artery aneurysm. These aneurysms have a high rate of rupture with severe bleeding and should be embolized. The use of embolization in cases with severe cirrhosis should be used with caution due to the dependence of arterial circulation in cirrhosis. The authors intended to embolize the aneurysm but there was a failure and the case took another turn. In fact, it became an example of endovascular grafting of a hepatic artery aneurysm. Iam sure this was of benefit for the patient because of the situation of cirrhosis and hopefully also eliminated the risk of later rupture.

Prof. B. W. Jeppsson

Department of Surgery

Malmö University Hospital

Malmö

S-205 02

Sweden

